# Omental lipoma in pediatric age group: clinical presentation, image findings and management

**DOI:** 10.31744/einstein_journal/2022RC5584

**Published:** 2022-02-08

**Authors:** Priscila Mina Falsarella, Antonio Rahal, Aline Andrade Dórea, Francisco Júlio Muniz, Miguel José Francisco, Rodrigo Gobbo Garcia, Marcos Roberto Gomes de Queiroz

**Affiliations:** 1 Hospital Israelita Albert Einstein São Paulo SP Brazil Hospital Israelita Albert Einstein, São Paulo, SP, Brazil.

**Keywords:** Omental lipoma/imaging diagnostic, Child

## Abstract

A 2-year-old female patient, admitted in the emergency room, presented diarrhea for 5 days and bloody stools in the last 24 hours. Physical examination revealed no significant findings. Ultrasound was initially performed, showing an elongated, well delimited and solid mass occupying since right hypocondrium until left iliac fossa, displacing adjacent structures. In sequence, magnetic resonance imaging was performed for confirmation of findings suggestive of omentum lipoma. After 1 week, the surgical resection was performed by videolaparoscopic acess. During 2-year follow-up, there was no sign of recurrence.

## INTRODUCTION

The greater omentum is formed by peritoneal folds derived from the mesogastrium, bounded by the great curvature of the stomach, transverse colon, proximal duodenum, and gastrosplenic ligament. It consists of adipose tissue, blood vessels, lymphatic vessels, and lymph nodes. At birth, these structures proliferate, and by the age of 5 years, the greater omentum resembles that of an adult individual. Primary tumors in this location are extremely rare. Among the differential diagnoses in this region, lipoma, desmoid tumor, fibroma, mesothelioma, leiomyoma, leiomyosarcoma, fibrosarcoma, and liposarcoma should be considered.^(1)^

Lipoma is a common condition in adults, although it is a rare presentation in children and adolescents, with only 6% of tumors in this age group; when observed 94% of lipoma are benign. The pathogenesis of this lesion is uncertain, however, in some reports in the published literature, there is an increased incidence associated with some factors: obesity, *diabetes mellitus*, elevated serum cholesterol, trauma, radiation, family history, and chromosomal abnormalities.^(2)^

This report describes a case of omentum lipomatosis as an incidental imaging finding in a pediatric patient.

## CASE REPORT

**Figure 1:**
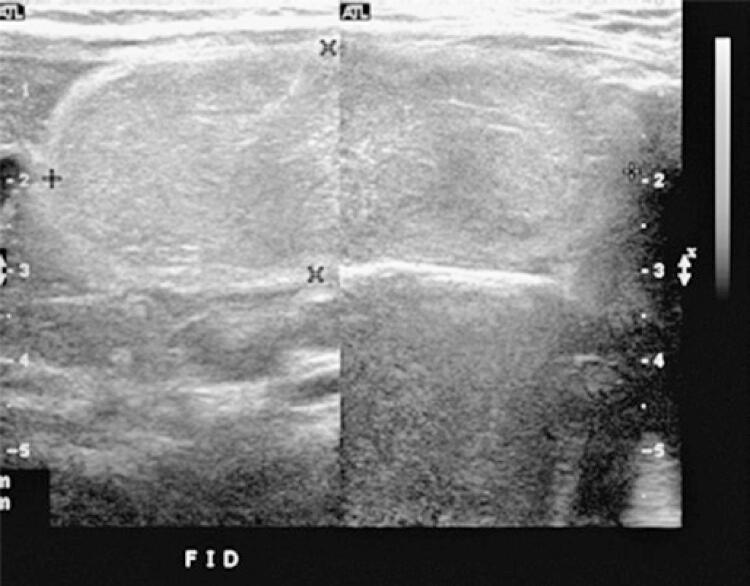
Ultrasonography showing an elongated, well-defined, echogenic and slightly heterogeneous mass, with low flow on Doppler assessment

A 2-year-old patient was admitted to the emergency room with diarrhea for 5 days and bloody stools for the past 24 hours, with no other clinical complaints. Physical examination revealed no signs of peritonitis or visceromegaly. Ultrasonography demonstrated an elongated, well-defined, echogenic and slightly heterogeneous mass (Figure 1), with rare flow sites at Doppler, measuring 6.5x2.5cm and extending from the right hypochondrium to the right iliac fossa, which was associated with a small amount of adjacent free fluid.

Despite the excellent characterization of the lesion by ultrasonography in the emergency department, the appearance was suggestive of lipoma or other histological variant, but not definitive to establish the correct treatment.

**Figure 2:**
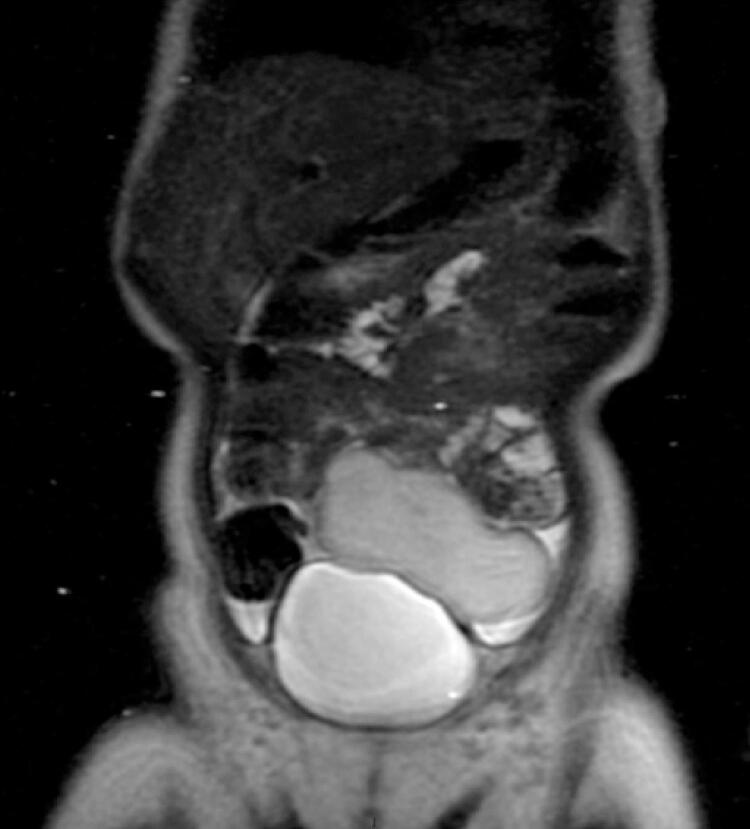
Coronal T2-weighted magnetic resonance imaging without gadolinium shows a mass with isosignal, discrete lobulated and well delimited, without infiltrative aspect, in close contact with the upper bladder wall and with the anterior surface of the peritoneum, displacing the upper intestinal loops and sigmoid colon, located in the meso/hypogastrium, extending to the left iliac fossa, and measuring 7.5x3.2x2.5cm

**Figure 3:**
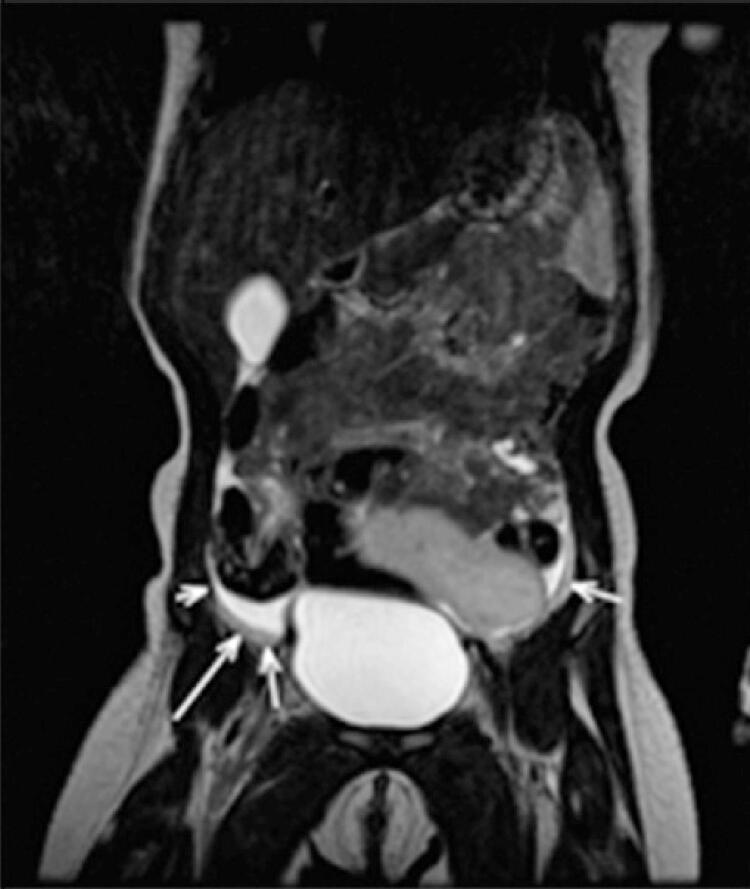
Coronal T2 magnetic resonance imaging sequence without gadolinium demonstrates small amount of free fluid in the pelvic cavity

**Figure 4:**
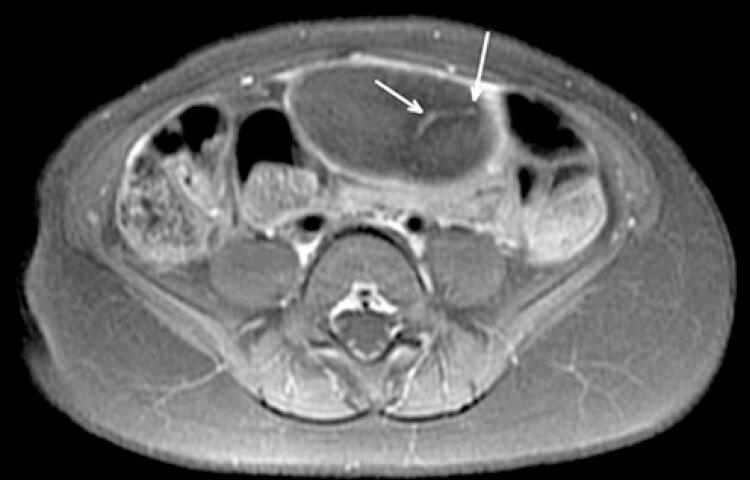
Magnetic resonance imaging, axial T1-weighted section, post-contrast with septum within the lesion and mild peripheral enhancement

Magnetic resonance imaging revealed a solid, slightly lobulated and well delimited mass, without infiltrative aspect, in contact with the upper bladder wall and anterior peritoneal surface, displacing the upper intestinal loops and sigmoid colon posteriorly. This was located in the meso/hypogastrium, extending from the right hypochondrium to the left iliac fossa (Figures 2 and 3), presented dimensions similar to ultrasonography, isosignal on T2-weighted sequence, and a discrete uptake on post-contrast T1-weighted sequence (Figure 4). In view of the imaging findings, with a well-defined mass and absence of other lesions or malignant factors, the diagnosis of omental lipoma was the main hypothesis.

The surgical approach was laparoscopic, under general anesthesia, using an infraumbilical incision and three portals (infraumbilical, 2 and 10cm from the inguinal ligament in the left hemiclavicular line). A tumor in the hypogastric was identified and its vessels were ligated with ultracision. A tumor mass 8.5x4.0x3.0cm in size was removed by infraumbilical incision, followed by aspiration of a small amount of free fluid in the pouch of Douglas. Pathological analysis established the diagnosis of omental lipoma. During the last 2 years of follow-up, and there was no sign of recurrence.

This study was approved by the Research Ethics Committee of *Hospital Israelita Albert Einstein* under number # 3.766.836, CAAE: 26505119.7.0000.0071.

## DISCUSSION

The evaluation of a child with an abdominal mass involves several diagnostic considerations. The possibilities considered depend on the child’s age, the location of the lesion, and the presence or absence of other related signs and symptoms. Determination of origin of the organ or tissue best guides the differential diagnostic possibilities.^(1)^

Omental lipomas are usually asymptomatic. Abdominal discomfort (45.5%), abdominal lump (34.9%) and abdominal distension (15.2%), nausea and weight loss may occur, occasionally. They are slow growing and may cause a sensation of a moving mass. Large lesions can cause distension and bowel obstruction. In these cases, they usually present as a palpable mass that is associated with pain. More rarely, they cause omental torsion and/or infarction. A careful clinical history, followed by a thorough physical examination, is extremely important in reaching a tentative diagnosis. Most cases are diagnosed only as incidental findings at laparotomy and autopsies. Incidental findings during an imaging study are also rare.^(1)^

Lipomas of the greater omentum are extremely rare, and the differential diagnosis of palpable abdominal mass in the pediatric age group is mainly nephroblastomatosis, Wilms’ tumor, neuroblastoma, hepatoblastoma, and other primary tumors of the greater omentum (lipoma, desmoid tumor, fibroma, mesothelioma, leiomyoma, leiomyosarcoma, fibrosarcoma, and liposarcoma).^(2-4)^

Ultrasound is widely available and extremely useful in establishing and excluding abdominal pathology. It is easy to perform, noninvasive and painless, and it requires no contrast material and excludes the risks of ionizing radiation. Ultrasonography, besides differentiating solid, cystic and complex lesions, helps to provide information about the site and organ of origin, the size and extent of the lesion, and invasion of adjacent organ systems.^(5,6)^

Omental lipomas are benign lesions, which usually appear on ultrasonography as a well-defined, hyperechogenic, homogeneous, non-calcified, avascular solid structure, or with a discrete flow on color Doppler mapping. However, sometimes they do not allow the identification of the site of origin as well as the precise relationship with adjacent structures. For this reason, another imaging method is required to guide the diagnosis.^(5-7)^

Magnetic resonance imaging is more specific to the evaluation of fatty lesions, such as lipomas, which consist of macroscopic fat. Although the diagnosis is usually strongly suggested by ultrasound and computed tomography scan, magnetic resonance imaging is better able to demonstrate the surrounding anatomy. They usually demonstrate an iso/hypersignal on T1 and T2 sequences, with absent or only minor uptake on post-contrast sequences.

Regarding treatment options, tumor resection and omentectomy are the most recommended, given that lipomas have low malignancy potential and small recurrence rates. After local excision, recurrence is less than 5%, often occurring due to the incomplete excision. In theory, there is no difficulty in enucleating and completely removing the lesion, and serious postoperative complications often do not occur.^(8)^

## CONCLUSION

Although omental lipomas are rare, they should be considered during ultrasonography as differential diagnosis. In cases of emergency evaluations, the ability to differentiate a malignant tumor from a benign condition is always fundamental.
